# Mayer-Rokitansky-Küster-Hauser Syndrome: A Case Report

**DOI:** 10.7759/cureus.67606

**Published:** 2024-08-23

**Authors:** Aarthi Muthu Kumar, Pramila Menon, Shailaja Mane

**Affiliations:** 1 Pediatrics, Dr. D. Y. Patil Medical College Hospital & Research Centre, Dr. D. Y. Patil Vidyapeeth (Deemed to be University), Pune, IND

**Keywords:** murcs, renal agenesis, uterovaginal aplasia, female patient, mullerian agenesis, mayer-rokitansky-küster-hauser (mrkh) syndrome

## Abstract

Mayer-Rokitansky-Küster-Hauser (MRKH) syndrome is described in females with a 46, XX karyotype and normal development of secondary sexual characteristics. The primary sexual characteristics are depicted by the congenital aplasia of the uterus and the upper two-thirds of the vagina. Based on the extent of malformations and association of extra-genital anomalies, it is categorized into type I and type II MRKH. The associated malformations seen include skeletal anomalies, renal anomalies, hearing defects, and, rarely, digital and cardiac anomalies. Herewith, we report a case of a two-year-old patient with urogenital anomalies on the left side diagnosed by imaging studies, which were suggestive of MRKH type II. For any child with urogenital anomalies with associated renal, skeletal, and hearing defects, we must suspect MRKH syndrome. The early detection of such anomalies will help in genetic counseling, management of fertility outcomes, and appropriate surgical management.

## Introduction

Mayer-Rokitansky-Küster-Hauser (MRKH) syndrome is defined by the congenital uterovaginal aplasia in females, who otherwise exhibit a normal 46, XX karyotype and typical development of secondary sexual characteristics [1]. Type I (isolated) MRKH syndrome is described as an isolated congenital aplasia of the uterus and the upper two-thirds of the vagina, which is also termed the Rokitansky sequence. When there is incomplete aplasia of the uterus and vagina coupled with other malformations, it is referred to as Müllerian duct aplasia-renal agenesis-cervicothoracic somite dysplasia (MURCS) association or type II MRKH syndrome [2]. These associated malformations often include renal anomalies (unilateral agenesis, ectopic kidneys, or horseshoe kidney), skeletal defects, particularly vertebral (Klippel-Feil anomaly, cervical fused vertebrae, and scoliosis), hearing defects, and, less commonly, cardiac and digital anomalies (syndactyly and polydactyly). The prevalence of these cases is approximately one in 5,000 births [3].

## Case presentation

A two-year-old girl, with an uneventful postnatal history born of a non-consanguineous marriage, was admitted for dengue fever. She presented with fever, abdominal distension, and intermittent dysuria. The child was fully immunized and developmentally appropriate for her age. General examination findings were normal with a weight of 9.9 kg and a height of 83 cm, and her external genitalia were characteristic of a prepubertal female. The investigations done for the patient are shown in Table 1.

**Table 1:** Biochemical investigations of the patient

Investigations	Patient values	Normal values
Haemoglobin (g/dl)	8	10.8-12.8
Total leukocyte count (cells/microliter)	10,400	4000-11000
Platelets (cells/mm^3_​​​​​​_^)	5,16,000	2,65,000-4,00,000
Urea (mg/dl)	51	5-18
Creatinine (mg/dl)	0.7	0.3-0.7
Dengue NS1 antigen	Positive	-
Urine microscopy	Increased pus cells	-

The patient initially had normal serum electrolytes but later developed hyperkalemia with a potassium level of 6.8 mmol/liter (normal serum potassium = 3.3-4.6mmol/L), which responded to medical management.

**Figure 1:**
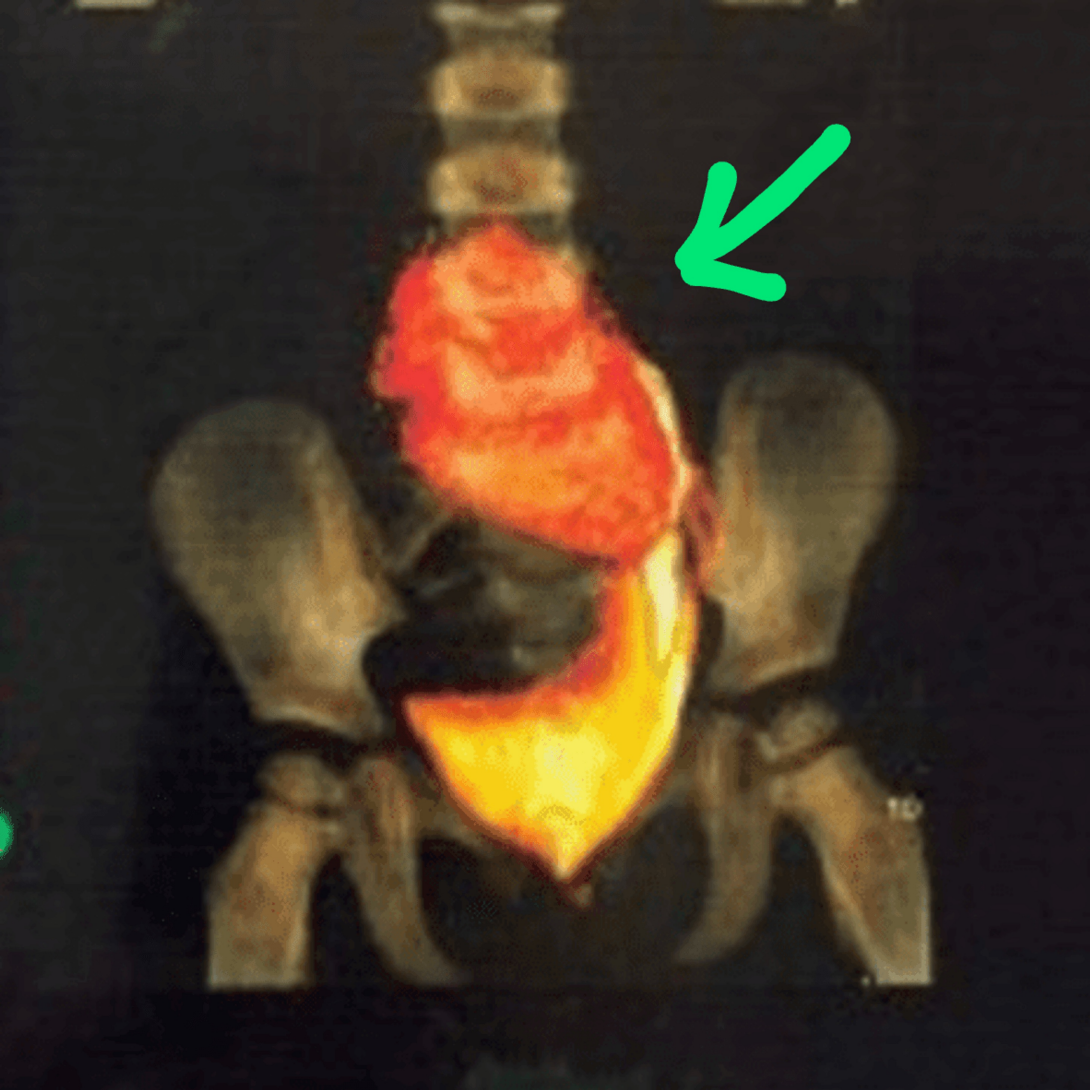
CT scan of the abdomen and pelvis showing the presence of a solitary right-sided ectopic kidney

**Figure 2:**
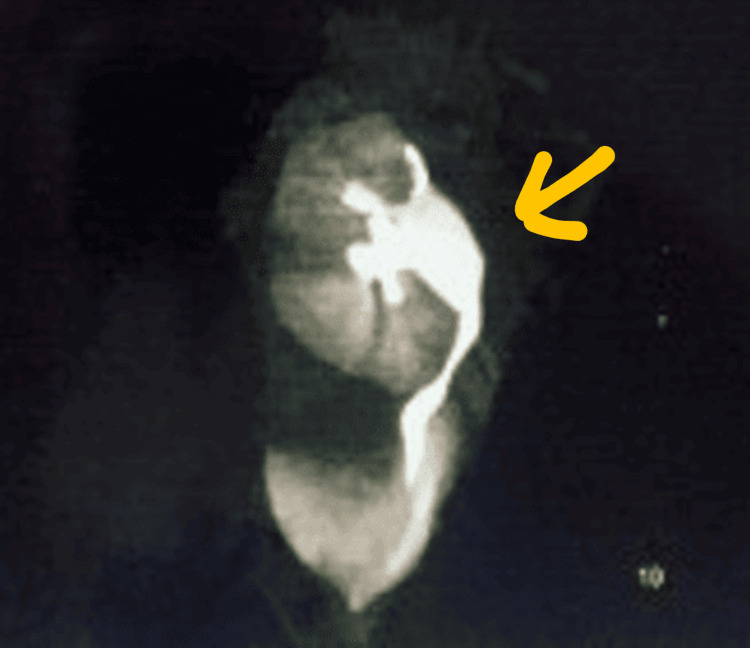
CT scan of the abdomen and pelvis showing the presence of a solitary right-sided ectopic kidney with a dilated extrarenal pelvis and right-sided hydroureter

Abdominal and pelvic ultrasonography indicated the absence of kidneys in the renal fossa. Magnetic resonance imaging (MRI) of the abdomen revealed a single midline ectopic kidney near the right border of the bladder. A radionucleotide scan assessed the kidney function, indicating a solitary, normally functioning, non-obstructed kidney. Micturating cystourethrography (MCU) showed grade 4 vesicoureteral reflux (VUR) on the right side with a right ectopic kidney. An echocardiogram ruled out associated anomalies, showing a normal heart, and karyotyping results were normal, ruling out Turner’s syndrome. A hormonal assay was not done in this case, which is a limitation in this report. A computed tomography (CT) scan as shown in Figure 1 confirmed the presence of a solitary right-sided ectopic pelvic kidney, and Figure 2 shows the right-sided ectopic kidney with a dilated extrarenal pelvis and right-sided hydroureter. The other findings noted in the CT scan were non-visualized uterus and ovaries, suggesting agenesis along with spina bifida occulta. These features are indicative of MRKH type II syndrome.

Diagnostic cystoscopy and laparoscopy confirmed normal vaginal opening with an absent lumen and bilateral normal ovaries. The uterus was streak-like with a normal right and absent left fallopian tube. A normal bladder with a single right ureteric opening was observed. Major urogenital abnormalities on the left side necessitate future ureteric reimplantation.

## Discussion

MRKH syndrome presents with aplasia in the uterus and vagina in females with normal ovaries, secondary sexual characteristics, and a typical 46, XX karyotype [1]. This condition arises from the abnormal development of the Müllerian ducts and the associated organs. MRKH closely resembles MURCS association, which is defined by abnormal sexual development, anomalies in cervicothoracic somite, unilateral renal agenesis, and conductive deafness [2]. MRKH syndrome is observed in approximately one in 5,000 births [3].

Skeletal anomalies, such as scoliosis, vertebral anomalies, rib malformations, and spina bifida, are present in 30-40% of cases [4]. In our patient, imaging studies identified spina bifida occulta. Auditory defects, including conductive deafness due to stapedial ankylosis or middle ear malformations and sensorineural defects, occur in 25% of MRKH type II cases [4]. Cardiac malformations are rare but may include atrial septal defects, pulmonary valvular stenosis, or tetralogy of Fallot [4]. While the exact cause of MRKH remains unknown, mutations in the WNT4 gene and elevated androgen levels have been associated with Müllerian aplasia [5]. Our patient, presenting with Müllerian agenesis, left kidney agenesis, and spina bifida occulta, exemplifies MRKH type II (MURCS) association.

The primary diagnostic tool for MRKH is imaging, with abdominal ultrasonography as the initial investigation and MRI of the abdomen and pelvis providing more detailed information [6].

This case report showed the early diagnosis of the patient at a relatively young age due to the associated anomalies. This helps in the early interventions and management of the anomalies with comprehensive management and follow-up in the long-term course.

Parent counseling is the first step done as it was a pediatric patient followed by the management of existing medical conditions, which was the priority in this case. Treatment involves both the non-surgical creation of neovagina and surgical creation of neovagina with counseling for options in the future with regard to fertility outcomes. Uterus transplant has been theorized as a treatment for infertility and has been successful in some cases. However, there are no standard guidelines for a therapeutic procedure, and more importantly, ethical issues need to be addressed [7].

## Conclusions

For any patient presented with Müllerian agenesis and associated anomalies like agenesis of the left kidney or spine abnormalities, we must suspect MRKH syndrome or variants like MRKH type II (MURCS) association. The other associated anomalies like cardiac, vertebral, and hearing defects should also be ruled out. Treatment involves the surgical correction of genital defects like the creation of neovagina and the correction of anomalies. Parental counseling for options in the future with regard to fertility outcomes is essential. Genetic diagnosis in MRKH is very important for offering genetic counseling to families.
